# Imaging phenotypes from MRI for the prediction of glioma immune subtypes from RNA sequencing: A multicenter study

**DOI:** 10.1002/1878-0261.13380

**Published:** 2023-02-12

**Authors:** Jingxian Duan, Zhenyu Zhang, Yinsheng Chen, Yuanshen Zhao, Qiuchang Sun, Weiwei Wang, Hairong Zheng, Dong Liang, Jingliang Cheng, Jing Yan, Zhi‐Cheng Li

**Affiliations:** ^1^ Institute of Biomedical and Health Engineering, Shenzhen Institute of Advanced Technology Chinese Academy of Sciences Shenzhen China; ^2^ Department of Neurosurgery The First Affiliated Hospital of Zhengzhou University Zhengzhou China; ^3^ Department of Neurosurgery/Neuro‐oncology, Sun Yat‐Sen University Cancer Center, State Key Laboratory of Oncology in South China Collaborative Innovation Center for Cancer Medicine Guangzhou China; ^4^ Department of Pathology The First Affiliated Hospital of Zhengzhou University Zhengzhou China; ^5^ Department of MRI The First Affiliated Hospital of Zhengzhou University Zhengzhou China; ^6^ National Innovation Center for Advanced Medical Devices Shenzhen China; ^7^ Shenzhen United Imaging Research Institute of Innovative Medical Equipment Shenzhen China

**Keywords:** glioma, immune escape, immune subtype, machine learning, radiogenomics

## Abstract

Tumor subtyping based on its immune landscape may guide precision immunotherapy. The aims of this study were to identify immune subtypes of adult diffuse gliomas with RNA sequencing data, and to noninvasively predict this subtype using a biologically interpretable radiomic signature from MRI. A subtype discovery dataset (*n* = 210) from a public database and two radiogenomic datasets (*n* = 130 and 55, respectively) from two local hospitals were included. Brain tumor microenvironment‐specific signatures were constructed from RNA sequencing to identify the immune types. A radiomic signature was built from MRI to predict the identified immune subtypes. The pathways underlying the radiomic signature were identified to annotate their biological meanings. The reproducibility of the findings was verified externally in multicenter datasets. Three distinctive immune subtypes were identified, including an inflamed subtype marked by elevated hypoxia‐induced immunosuppression, a “cold” subtype that exhibited scarce immune infiltration with downregulated antigen presentation, and an intermediate subtype that showed medium immune infiltration. A 10‐feature radiomic signature was developed to predict immune subtypes, achieving an AUC of 0.924 in the validation dataset. The radiomic features correlated with biological functions underpinning immune suppression, which substantiated the hypothesis that molecular changes can be reflected by radiomic features. The immune subtypes, predictive radiomic signature, and radiomics‐correlated biological pathways were validated externally. Our data suggest that adult‐type diffuse gliomas harbor three distinctive immune subtypes that can be predicted by MRI radiomic features with clear biological significance. The immune subtypes, radiomic signature, and radiogenomic links can be replicated externally.

## Graphical abstract

We developed a brain‐specific tumor microenvironment gene signature for the identification of glioma immune subtypes, and established a radiomic signature that allows the prediction of glioma immune subtype using MR images. This noninvasive method enables the surveillance of immunological dynamics of gliomas throughout the course of the disease.

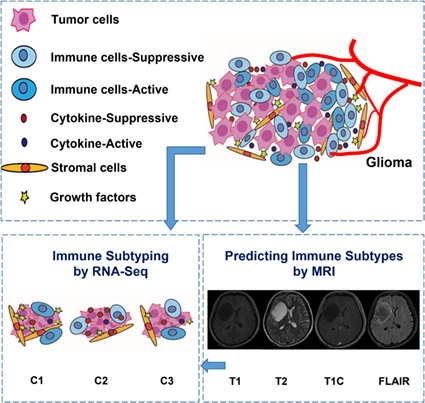

**Abbreviations**
AUC: area under the receiver operating characteristic curve
GBM: glioblastomas
ICC: intraclass correlation coefficient
IGP: in‐group proportion
RG: radiogenomic
RNA‐seq: RNA sequencing
ROC: receiver operating characteristic
SD: subtype discovery
ssGSEA: single sample gene set enrichment analysis
TIL: infiltrating lymphocytes
TME: tumor microenvironment
VOI: volumes of interest

## 1 Introduction

Diffuse gliomas are the most frequent brain tumors in adults and have been historically classified into WHO grade 2–4 according to their histological features [1, 2]. Despite multimodal treatments including surgical resection, chemotherapy, and radiotherapy, most diffuse gliomas inevitably recur at later stages and are likely to transform into or behave like glioblastomas (GBM), the most malignant WHO grade 4 gliomas [3]. GBM has a median survival of merely 15.4 months despite aggressive therapies [4], and always exhibit progressive treatment resistance [5]. Recently, the WHO classification has been improved into integrated categories by incorporating molecular markers such as the isocitrate dehydrogenase (IDH) mutation and 1p/19q codeletion, aiming to align with the emergence of targeted therapies [6]. Still, pioneer studies are required to explore the feasibility of new therapies with the corresponding tumor subtyping.

Immunotherapy has achieved remarkable success in multiple types of cancer. However, immunotherapy has shown significant clinical benefits only in a subset of patients [7, 8, 9]. The lack of tumor infiltrating lymphocytes (TILs) in the brain tumor microenvironment (TME) is likely the main contributor to the “immune‐cold” phenotype of gliomas, which leads to inadequate immunotherapy responsiveness. However, subsets of GBM were shown to respond to immunotherapy because they exhibited a TIL enriched “immune‐hot” TME [10, 11, 12]. Identifying glioma immune subtypes by calculating TIL abundance could facilitate precision immunotherapy in glioma. In this regard, transcriptomic‐based TIL estimation algorithms including Cibersort, xCell, TIMER, quanTIseq, EPIC, and MCP‐counter were widely applied to calculate TIL abundance in tumors [13, 14, 15, 16]. However, these pan‐cancer algorithms did not include gene signatures for the estimation of microglia, the brain resident immune cell that is exclusive to glioma. Due to the ignorance of the key immune modulator of glioma, current methods cannot reliably categorize the immune subtype of adult‐type diffuse gliomas.

Transcriptome‐based molecular subtyping of GBM has been proposed to inform personalized treatment [17, 18]. The molecular features of tumors change dynamically during the course of treatment. However, RNA sequencing (RNA‐seq) can only be performed after tumor tissues were resected. It is not possible to obtain tumor tissue samples repetitively for the real‐time monitoring of these molecular alterations. Moreover, the invasive operation shortened the time window within which the treatment regimen could be adjusted accordingly, and restricted the application of personalized neoadjuvant therapy in gliomas [19]. Radiomics enables noninvasive characterization of glioma phenotypes by retrieving quantitative features from imaging. It has been applied to predict patient survival and treatment outcomes [20, 21, 22, 23]. Predicting glioma immune subtypes with radiomic features allows the identification of glioma immune subtypes at diagnosis, and surveillance of the dynamic changes in the glioma TME along the course of the disease. However, the lack of biological meaning and multicenter reproducible evidence posed a clear obstacle to the clinical application of radiomics.

Therefore, the purposes of the study were to identify immune subtypes of adult‐type diffuse gliomas with brain TME‐specific immune signatures from RNA‐seq, and to predict the immune subtypes noninvasively using radiomics from MRI. Furthermore, we also assessed whether the immune subtypes, the radiomic signature, and the radiogenomic linkage between the radiomics features and underlying biological pathways can be replicated in multicenter datasets.

## 2 Materials and methods

### 2.1 Study design

This retrospective multicenter study included four major steps: immune subtype discovery, radiomic signature building, radiomic‐associated pathways identification, and validation of reproducibility. First, we identified immune subtypes from RNA‐seq using a transcriptome‐based immune subtyping method. Second, we built a radiomic signature from MRI to predict immune subtypes noninvasively. We then identified pathways underpinning the radiomic features to uncover the biological meaning of the radiomic signature. Lastly, we validated the reproducibility of the identified immune subtypes, the predictive value of the radiomic signature, and the biological interpretability of the radiomic signature on external datasets.

### 2.2 Datasets

**Fig. 1:**
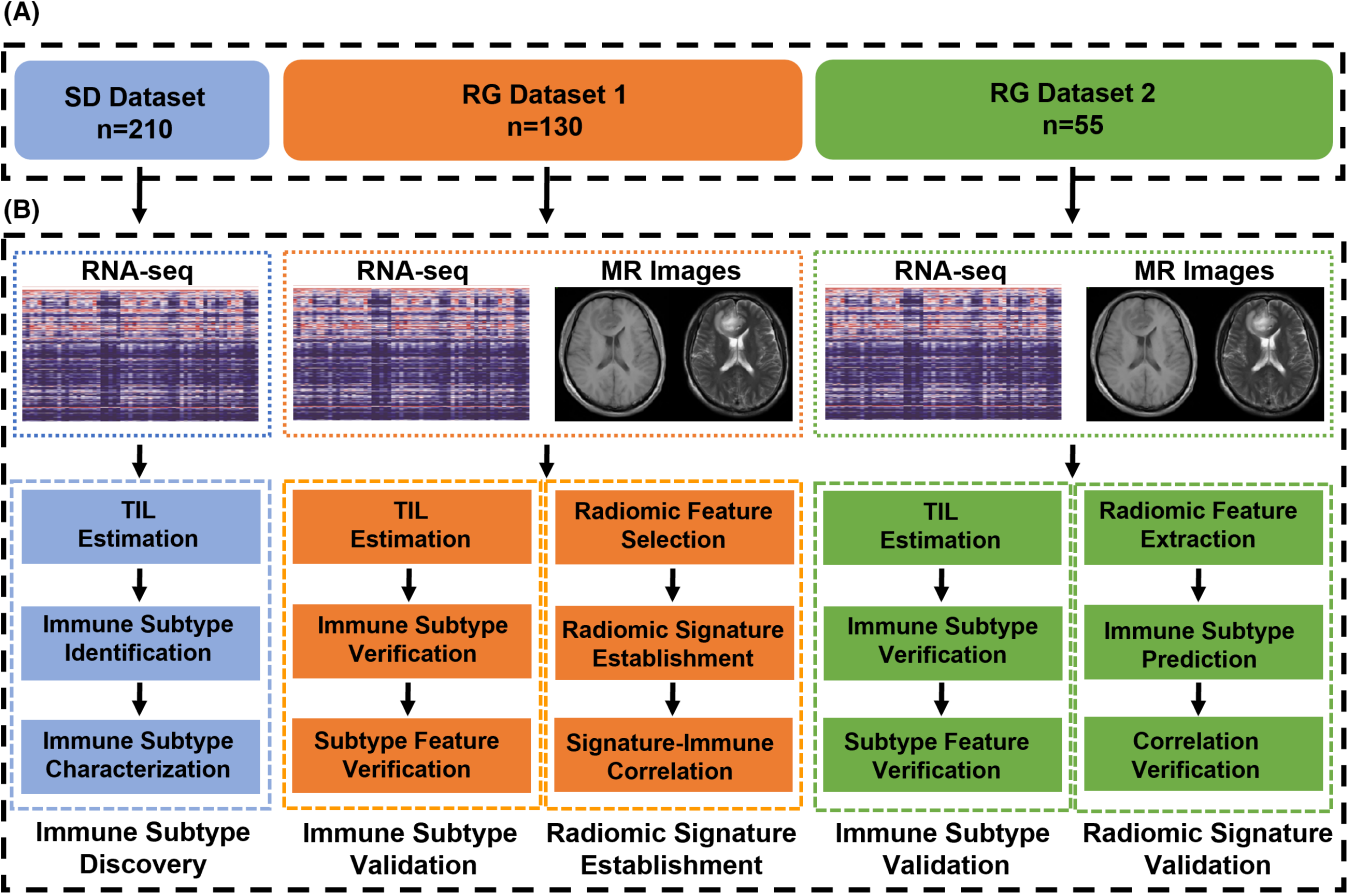
Overview of the study. (A) Patient cohorts included in the study. (B) Data type and overall design of the study. MR, magnetic resonance; TIL, tumor infiltrating lymphocytes.

This study was approved by the Human Scientific Ethics Committee of the First Affiliated Hospital of Zhengzhou University (FAHZZU) and the Sun Yat‐Sen University Cancer Center (SYSUCC). The study comprised three datasets: (a) a subtype discovery (SD) dataset including 210 patients with RNA‐seq data collected from the CGGA database for identifying immune subtypes; (b) a radiogenomic (RG) dataset 1 containing 130 patients with both MRI and RNA‐seq collected from FAHZZU between January 2012 and December 2018 for replicating the identified immune subtypes and for developing a radiomic signature with underlying biological pathways; and (c) an RG dataset 2 containing 56 patients with paired MRI and RNA‐seq collected from SYSUCC between January 2015 and December 2016 to further replicate the immune subtypes and to validate the prediction performance, as well as the underlying biological pathways of the radiomic signature (Fig. 1). Patients with adult diffuse gliomas were recruited, comprising WHO grade 2–4. Written informed consent was obtained for both RG datasets. The patients were followed‐up by telephone or clinic every 3–6 months. They received radiation therapy and/or chemotherapy, as illustrated in Table 1. Survival was measured as the interval between the date of initial pathologic diagnosis and the date of death/progression or the end of follow‐up. Pathological images of the tumor specimens were also collected if available. The patient inclusion and exclusion criteria for the three datasets are provided in Table S1.

**Table 1:** Patient demographics

Characteristics	Subtype discovery dataset (*n* = 210)	Radiogenomic dataset 1 (*n* = 130)	Radiogenomic dataset 2 (*n* = 55)	*P*‐value
Age (years)	49.3 ± 0.9	49.6 ± 1.2	50.3 ± 1.7	0.9 (3)
Gender				
Male	117 (55.7%)	72 (55.4%)	30 (53.6%)	—
Female	91 (43.3%)	58 (44.6%)	25 (44.6%)	—
WHO grade				
2	48 (22.9%)	29 (22.3%)	14 (25%)	—
3	22 (10.5%)	16 (12.3%)	4 (7%)	—
4	140 (66.7%)	85 (65.4%)	37 (66.1%)	—
IDH				
Mutant	73 (34.8%)	51 (39.2%)	19 (33.9%)	—
Wildtype	130 (61.9%)	79 (60.8%)	36 (64.3.1%)	—
1p19q				
Codeleted	35 (16.7%)	22 (17.0%)	13 (23.2%)	—
Noncodeleted	148 (70.5%)	108 (83.1%)	42 (75.0%)	—
MGMTp				
Methylated	93 (44.3%)	—	—	—
Nonmethylated	80 (38.1%)	—	—	—
TERT promoter				
Mutant	—	72 (55.4%)	34 (60.7%)	—
Wildtype	—	57 (43.8%)	20 (35.7%)	—
Radiation therapy				
Yes	163 (77.6%)	84 (64.6%)	34 (60.7%)	—
No	34 (16.2%)	46 (35.4%)	22 (39.3%)	—
Chemotherapy				
Yes	157 (74.8%)	103 (79.2%)	43 (76.8%)	—
No	39 (18.6%)	27 (20.8%)	13 (23.2%)	—
OS (months)	35.2 ± 2.3	17.6 ± 0.6	15.9 ± 1.2	< 0.001 (3) 0.26 (2)
PFS (months)	—	15.7 ± 1.2	15.7 ± 1.9	0.83 (2)

Unless otherwise noted, data are numbers of patients, with percentages in parentheses. Age, overall survival (OS), and progression‐free survival (PFS) are represented as means ± standard deviations. *P* value is calculated for the difference in patient characteristics between three datasets (noted as 3), or between the training and validation datasets (noted as 2). IDH, isocitrate dehydrogenase.

### 2.3 RNA sequencing data acquisition and preprocessing

RNA sequencing was performed using pretreatment tumor tissue samples, which were collected during primary surgery from patients in RG datasets 1 and 2. Raw data were processed with the fastq standard protocol [24], including quality control, adapter trimming, quality filtering, and per‐read quality cutting. Paired‐end clean reads were aligned to the reference genome using hisat2 v2.1.0 (Dallas, Texas, U.S.). Reads mapped to each gene were defined as feature counts, and FPKM (expected number of fragments per kilobase of transcript sequence per millions base pairs sequenced) of each gene was calculated. The average sequencing depth of the SD dataset was 30.3 ± 0.3 million reads per sample. The average sequencing depths of RG datasets 1 and 2 were 29.7 ± 0.3 million reads per sample and 29.8 ± 0.4 million reads per sample, respectively (Fig. S1A).

### 2.4 MR images acquisition and preprocessing

For each patient in RG datasets 1 and 2, preoperative MRI were collected, including T1‐weighted, T2‐weighted, T2‐weighted FLAIR, and T1‐weighted gadolinium contrast‐enhanced imaging (T1, T2, FLAIR, and T1c, respectively).

All patients in FAHZZU were performed on either 1.5T or 3.0T clinical MR scanners. The 1.5T scanners included Philips Healthcare (Achieva, Best, The Netherlands). The 3.0T scanners included Siemens Healthcare (Magnetom Skyra/Prisma/Trio TIM/verio, Erlangen, Germany), GE Healthcare (Discovery MR750/MR750w/Signa HDxt, Milwaukee, WI, USA), and Philips Healthcare (Ingenia, Best, The Netherlands). The brain imaging protocol includes the following sequences: (a) axial T1; (b) axial T2; (c) axial T2‐weighted FLAIR; and (d) axial T1c. The contrast‐enhanced sequences were acquired immediately after intravenous administration of a 0.1 mmol·kg^−1^ dose of gadolinium‐based contrast agent (Gadopentetic Acid Dimeglumine Salt Injection, Magnevist, Bayer Healthcare, Berlin, Germany, or Gadoteric Acid Meglumine Salt Injection, Hengrui Healthcare, Jiangsu, China), followed by a 20‐mL saline flush with an injection velocity of 2.0 mL·s^−1^.

All patient studies in SYSUCC were performed on either 1.5T or 3.0T clinical MR scanners. The 1.5T scanners included Philips Healthcare (Achieva). The 3.0T scanners included Siemens Healthcare (Magnetom Skyra/Prisma/Trio TIM/verio), GE Healthcare (Discovery MR750/MR750w/Signa HDxt), Philips Healthcare (Ingenia), and United Imaging (uMR780, Shanghai, China). The brain imaging protocol includes the following sequences: (a) axial T1; (b) axial T2; (c) axial T2‐weighted FLAIR; and (d) axial T1c. The contrast‐enhanced sequences were acquired immediately after intravenous administration of a 0.1 mmol·kg^−1^ dose of contrast agent (Gadopentetic Acid Dimeglumine Salt Injection, Magnevist, Bayer Healthcare, Berlin, Germany, or Gadodiamide, Omniscan, GE Healthcare, Princeton, NJ, USA), followed by a 20‐mL saline flush with an injection velocity of 2.0 mL·s^−1^.

All images were preprocessed to normalize the intensity and geometry. First, N4ITK was applied to correct bias field distortion [25]. Next, all voxels were isotropically resampled into 1 × 1 × 1 mm^3^ with linear interpolation for consistent feature extraction. The four MR sequences were rigidly registered using axial resampled T1c as a template. Histogram matching was used for intensity normalization. Three‐dimensional volume of interest (VOI) of tumor contours were manually delineated slice‐by‐slice by a neuroradiologist (J.Y. with 8 years of experience) and a neurosurgeon (Z.Y.Z. with 11 years of experience), blinded to clinical data. The VOI was drawn with the ITK‐SNAP software in the axial plane of the FLAIR sequences. T2 and T1c sequences were used to cross‐check the extension of the tumor and fine‐tune the tumor contour.

Fifty patients were randomly selected, which generated an intrarater test set and an interrater test set. The intrarater and interrater repeatability of a feature was assessed by the intraclass correlation coefficient (ICC) computed on the two test sets, respectively. Any feature with ICC < 0.85 was discarded.

### 2.5 Identification of glioma immune subtypes

To identify the immune subtypes of gliomas from RNA‐seq data using the SD dataset, we first estimated single‐sample TIL levels using a transcriptomic‐based algorithm. Published immune gene signatures [26, 27] and gene markers for microglia [28, 29] were combined to build a gene signature panel that was tailored to estimate the microenvironment of gliomas. It comprised 803 marker genes curated from the published literature [27, 28, 29], and can be applied to calculate the relative abundance of 29 immune cell types. Marker genes that were shown to distinguish microglia from macrophages/monocytes in glioma single‐cell RNA studies were included to form a glioma‐specific immune gene panel (Table S2a). Single‐sample gene set enrichment analysis (ssGSEA, GSVA function in r) was applied to calculate the abundance of each immune cell type with RNA expression data [30]. CIBERSORT [31] and xCell [15] were applied to validate the estimated immune infiltration. The tumor score, immune score, and microenvironment score of the samples were obtained using the estimate [32] r package.

In the SD dataset, the samples were clustered using the K‐means clustering algorithm (kmeans function in r), where 1000 iterations were performed and 80% of the input was sampled per iteration. As shown in Fig. S1B, the optimal cluster number was determined using the nbclust r package [33]. The stabilized K‐means model with refined centroids was used to determine the immune subtype of patients, and was used for patient stratification in survival analysis. A heatmap was plotted to show the cluster distribution.

### 2.6 Characterization of glioma immune subtypes

In the SD dataset, differential expression analysis of three immune subtypes was performed using the deseq r package (v. 1.18.1). The resulting *P*‐values were adjusted using the Benjamini and Hochberg's approach to control the false discovery rate (FDR). Genes with log2 (FoldChange) > 1, FDR < 0.05, and adjusted *P* < 0.001 were assigned as differentially expressed. Gene Ontology (GO) Biological Process, Kyoto Encyclopedia of Genes and Genomes (KEGG), and Reactome enrichment analysis of DEGs were performed using the clusterProfiler R package [34]. Pathways showing an adjusted *P* < 0.0001 and FDR < 0.05 were significantly enriched and were selected for downstream analysis. Gene set enrichment analysis (GSEA) was performed using the gsea r package [30], where hallmark gene sets, ontology gene sets, and immune escape gene sets were obtained from the molecular signatures database (MSigDB) [35]. Gene signatures for angiogenesis and ECM remodeling were adapted from the published literature [26] and the MSigDB, where the list of the gene sets can be found in Table S2b‐d. PD‐1 signaling and CTLA‐4 inhibitory signaling participants were downloaded from the Reactome database. The immune checkpoint (ICP) blockade protein list was obtained from the published literature [36].

### 2.7 Establishment of radiomic signature for predicting immune subtypes

In RG dataset 1, we aimed to construct a radiomic signature from preoperative MRI to noninvasively predict the identified immune subtypes. The radiomic signature training and radiomic feature selection were performed exclusively on RG dataset 1, which is independent of RG dataset 2. Radiomic features were extracted from the VOIs of each MR sequence using the pyradiomics package implemented in Python [37]. A total of 851 features were extracted per patient, including 14 shape features, 18 intensity features, and 75 texture features in original images (107 features) and wavelet transformed images (744 features). Descriptions of the radiomic features can be found in Table S3. Variations in feature values caused by different MR scanners were harmonized using the Combat algorithm provided by the sva r package [38].

Feature selection was performed to select an optimal subset of features. First, to select repeatable features against the delineation variations, intrarater and interrater repeatability of each feature was assessed by the intraclass correlation coefficient (ICC) computed on the two test sets, respectively. Any feature with ICC < 0.85 was discarded. Then, one‐way ANOVA or Kruskal–Wallis test was applied to examine the statistical difference of each feature among the three immune subtypes. Features having statistical differences (*P* < 0.05) across subtypes were selected. Based on the selected features, a random forest classifier with 100 trees was trained on RG dataset 1. According to the criteria for feature number selection in the published literature [20], the top 10 radiomic features were selected based on feature importance. A new random forest classifier was trained on the 10 radiomic features, yielding a radiomic signature for predicting the immune subtypes. The performance of the classifier was assessed in terms of accuracy, area under the receiver operating characteristic (ROC) curve (AUC), sensitivity, specificity, and F1 score on both the training set (RG dataset 1) and a validation set (RG dataset 2). The feature maps showing the visual properties of the 10 features used to constitute the signature were calculated.

### 2.8 Identification of biological functions underlying radiomic phenotypes

To explore the biological meanings of the 10 features that formed the radiomic signature, we sought to identify the pathways associated with each of these features. In RG dataset 1, Pearson's correlation coefficients between the enrichment score of the pathways and features in the radiomics signature were calculated. The *P* values were adjusted with the Benjamini and Hochberg's approach using the *P*.adjust function in r. Adjusted *P* < 0.05 was considered significant. The correlations between the estimated TIL and signature features were also calculated with the method described above.

### 2.9 Reproducibility validation of the identified subtypes and pathways

The reproducibility of the identified immune subtypes, the subtype characteristics, and the radiomics‐associated pathways were validated externally. First, we assessed whether the immune subtypes identified in the SD dataset can be replicated in both RG datasets 1 and 2. As in a previous publication [39], we computed the in‐group proportion (IGP) to measure the reproducibility of clusters across the discovery and validation sets. A K‐means classifier and centroids build on the SD dataset were applied to predict cluster in RG datasets 1 and 2. IGP was calculated as the proportion of samples whose prediction fell into their true classification. Second, we evaluated whether the immune subtypes in RG datasets 1 and 2 exhibited molecular characteristics consistent with those of the SD dataset. Third, we examined the predictive value of RG dataset 1 derived radiomic signature in RG dataset 2. Lastly, we assessed whether the radiomics‐associated biological pathways identified in RD dataset 1 could appear again in RD dataset 2.

### 2.10 Statistical analysis

All statistical analyses were performed using r (v. 4.1.0), python (v. 3.8), or graphpad prism (v. 9.3.0 for Windows, GraphPad Software, San Diego, CA, USA). The normality of each dataset was assessed; datasets that passed the normality test were compared with standard one‐way ANOVA or Kruskal–Wallis test depending on the result of the variation test. The datasets that did not display normal distribution were compared by the Kruskal–Wallis test. Multiple comparisons between the three immune subtypes were conducted by Dunnett's multiple comparisons test.The Pearson's correlation coefficient was calculated with python. *P* < 0.05 was considered statistically significant.

### 2.11 Ethical statement

The studies involving human participants were reviewed and approved by the Ethics Committee of the First Affiliated Hospital of Zhengzhou University (2019‐KY‐176) and the Sun Yat‐Sen University Cancer Center (SZR2020‐073). The study methodologies conformed to the standards set by the Declaration of Helsinki. The experiments were undertaken with the understanding and written consent of each patient.

## 3 Results

### 3.1 Patient demographics and datasets allocation

A total of 395 patients with histologically confirmed gliomas were enrolled, comprising 210 patients in the SD dataset, 130 patients in RG dataset 1, and 55 patients in RG dataset 2 (Fig. 1). The mean age of the patients in the three datasets showed no statistical difference (49.3 ± 0.9 years, 49.6 ± 1.2 years, and 50.4 ± 1.7 years, respectively, *P* = 0.90). Variations of gender ratio, tumor grade ratio, IDH mutation rate, 1p19q codeletion rate, and TERT promoter mutation rate were <6.2% among the three datasets (Table 1). Notably, patients in the SD dataset showed a significantly longer overall survival (OS, 35.2 ± 2.3 months *vs* 17.6 ± 0.6 months for RG dataset 1 and 15.9 ± 1.2 months for RG dataset 2, *P* < 0.001), because they had longer follow‐up (158 months for the SD dataset, 38 months for RG dataset 1, and 29 months for RG dataset 2). However, RG dataset 1 and RG dataset 2 showed no statistical difference in OS and progression‐free survival (PFS).

### 3.2 Glioma immune subtypes characterized by distinct TIL were identified

**Fig. 2:**
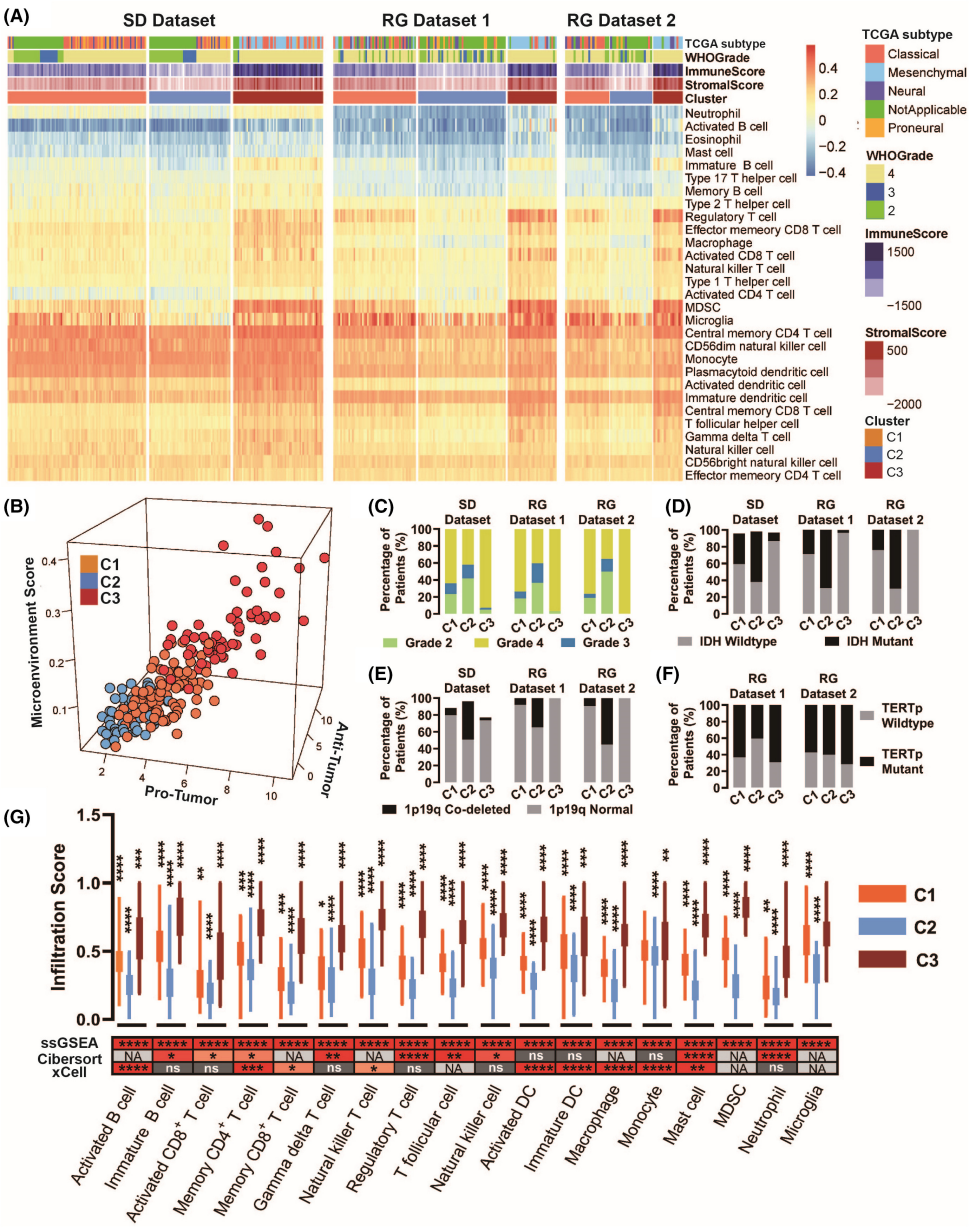
The three distinct immune subtypes identified in glioma. (A) Heatmap showing glioma patients were classified into three distinct immune subtypes based on the unsupervised clustering of the estimated infiltration level of 29 immune cells. The same analyses were performed on the SD dataset, RG dataset 1, and RG dataset 2, which yielded the same three subtypes. The subtypes are color coded (orange for C1, blue for C2, and brick for C3). The TCGA molecular subtype (classical, red; mesenchymal, steel blue; neural, dark orchid; proneural, light orange; low‐grade gliomas that were not applicable, green), WHO grade (Grade 4, bisque; Grade 3, navy; Grade 2, grass green), immune scores, and stromal scores were annotated for each sample. The immune scores and stromal scores were calculated with the ESTIMATE R package. MDSC, myeloid‐derived suppressor cells. (B) 3D scattered plot showing the protumor score, antitumor score, and microenvironment score of the three immune subtypes. The protumor score was the mathematical sum of normalized infiltration scores of MDSCs, regulatory T cells, neutrophils, and M2 macrophages. The antitumor score was the mathematical sum of normalized infiltration scores of activated CD4^+^ T cells, activated CD8^+^ T cells, activated B cells, natural killer cells, and M1 macrophages. The microenvironment scores were calculated with the xCell R package. (C) The percentage of patients that harbored grade II, III, and IV gliomas in the SD dataset, RG dataset 1, and RG dataset 2. (D) The percentage of patients who harbored the IDH mutant or IDH wildtype gliomas in the SD dataset, RG dataset 1, and RG dataset 2. (E) As in (D), but for 1p19q codeletion. (F) The percentage of patients harbored TERTp mutated or TERTp wildtype gliomas in RG dataset 1 and RG dataset 2. (G) Boxplots showing the infiltration scores of 18 types of immune cells that were differentially enriched among the three immune subtypes. The upper and lower limits represented the minimum and maximum values, whereas the first and third quartiles are shown by a box. Statistical comparisons were conducted with one‐way ANOVA or Krustal–Wallis test, depending on the normality of the datasets. *P* values were calculated with the chi‐square test. *P < 0.05, **P < 0.01, ***P < 0.001, ****P < 0.0001. *n* = 94 for C1, *n* = 55 for C2, and *n* = 61 for C3. * Above orange boxes show the comparison between C1 and C2, * Above blue boxes show comparison between C2 and C3. * Above brick boxes show comparison between C1 and C3. The immune cell infiltration was calculated by ssGSEA, Cibersort, and xCell separately; the *P* values resulting from statistical comparisons between the three subtypes are displayed in the heatmap.

To classify the immune landscape of glioma using transcriptomic‐based analytical methods, the relative abundances of 29 types of immune cells were calculated using a customized immune signature set (Fig. 2A). We applied an immune deconvolution method that was used to identify conserved pan‐cancer microenvironment subtypes [26], which could detect conserved immune subtypes in adult diffuse gliomas. By performing unsupervised clustering on the abundance of TILs in the SD dataset, we identified three glioma immune subtypes that exhibited distinctive immune cell infiltration levels. Subtype C1 showed medium infiltration of both protumor and antitumor immune cells; subtype C2 appeared to represent ”cold” tumors that exhibited scarce immune cell infiltration; while the inflamed subtype C3 was marked by evidently higher infiltration of both protumor and antitumor immune cells (Fig. 2B).

All three immune subtypes harbored grade 2, 3, and 4 gliomas (Fig. 2C), IDH mutant gliomas (Fig. 2D), 1p19q codeleted gliomas (Fig. 2E), and TERT promoter mutated gliomas (Fig. 2F). The immune subtypes were distributed across all WHO grades and molecular groups, suggesting that the immune subtypes represented a conserved classification across all types of adult‐type diffuse glioma. In great contrast, the expression levels of 27 immune cell types were significantly different among the three immune subtypes (93.1%, *n* = 29, *P* < 0.05). To validate the difference in immune cell infiltration, we recalculated the cell‐type enrichment scores with xCell [15] and Cibersort [14]. Excluding five cell types that cannot be estimated by either method, the statistical differences in TIL between the three immune subtypes were confirmed by xCell or Cibersort in 16 types of immune cells (Fig. 2G), including T cells, B cells, mast cells, macrophages, NK cells, microglia, myeloid‐derived suppressor cells, dendritic cells, and neutrophils.

### 3.3 Glioma immune subtypes were characterized by varied immune escape mechanisms

**Fig. 3:**
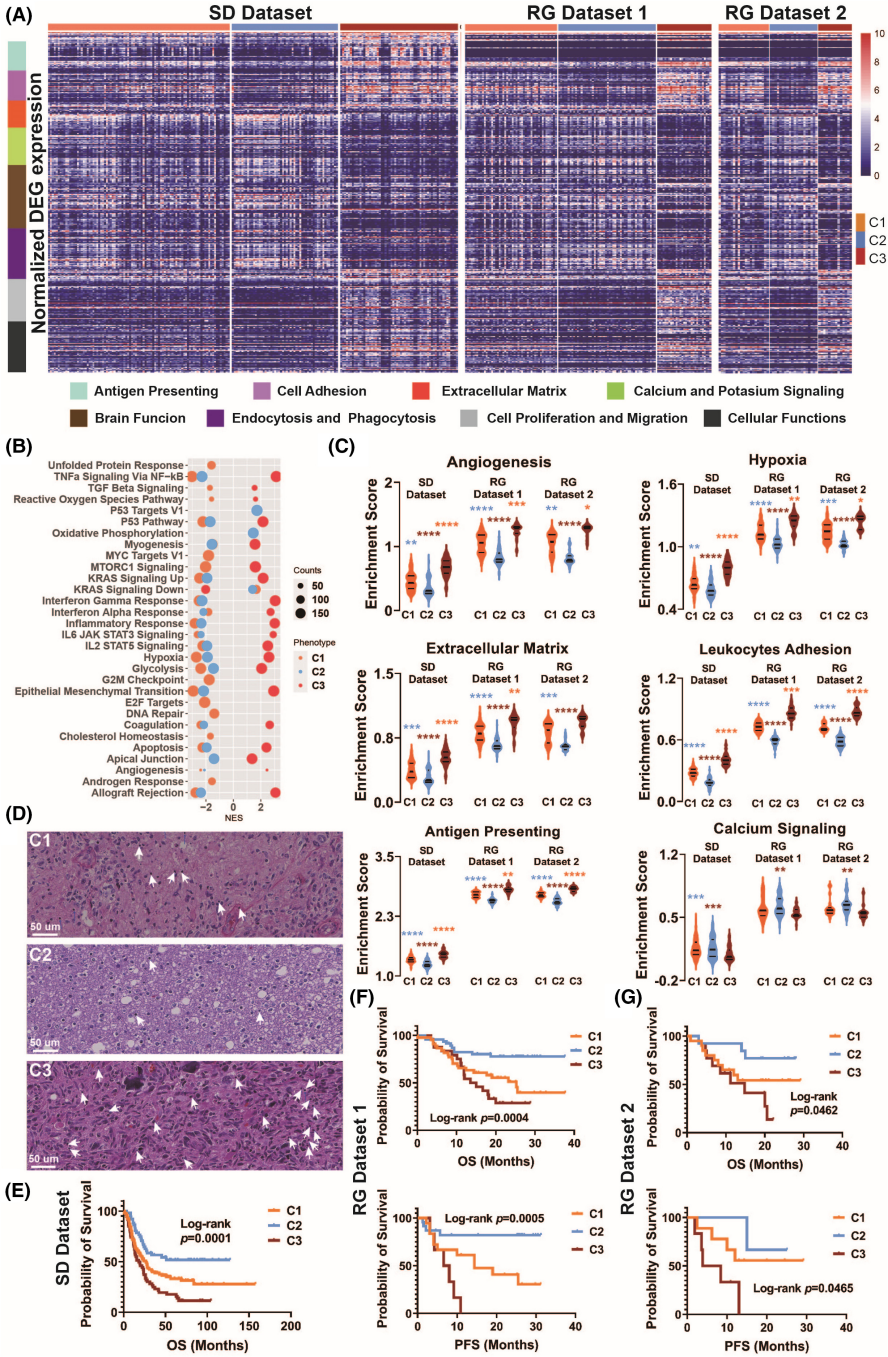
The three immune subtypes applied different immune escape strategies and showed distinct patient prognoses. (A) Heatmap showing the differentially expressed genes between tumors of one immune subtype versus the rest based on RNA abundance. DEGs were screened by FDR < 0.05, adjusted *P* < 0.001, and log2 (FoldChange) > 1. The DEGs were computed in the SD dataset, and were also plotted in RG dataset 1 and RG dataset 2. DEG enriched signaling pathways were labeled by color bars. Pathways showing the adjusted *P* value < 0.0001 and FDR < 0.05 were included. (B) The normalized enrichment scores of the three subtypes for GSEA hallmark gene sets are shown in the bubble plot. The size of the bubbles denotes the counts of genes significantly enriched for each pathway. The subtypes were color‐coded (orange for C1, blue for C2, and brick for C3). (C) Violin plots showing the ssGSEA enrichment scores for angiogenesis, extracellular matrix remodeling, antigen presenting, hypoxia, leukocytes adhesion, and calcium signaling of the three immune subtypes in the SD dataset (*n* = 94 for C1, *n* = 55 for C2, and *n* = 61 for C3), RG dataset 1 (*n* = 49 for C1, *n* = 29 for C2, and *n* = 52 for C3), and RG dataset 2 (*n* = 21 for C1, *n* = 20 for C2, and *n* = 14 for C3). Statistical comparisons were conducted with one‐way ANOVA or Krustal–Wallis test depending on the normality of the datasets. *P* values were calculated with the chi‐square test. *P < 0.05, **P < 0.01, ***P < 0.001, ****P < 0.0001. (D) Representative H&E‐stained histological images of the three immune subtypes. Examples of immune cells are indicated by white arrows. Scale bars denote 50 μm. (E) Kaplan–Meier curves showing the probability of patient survival in three immune subtypes, calculated using the SD dataset. Number of patients at risk table was attached below the curves. (F) Kaplan–Meier curves comparing the OS (upper panel) and radiation therapy PFS (lower panel) of the three immune subtypes in RG dataset 1. Number of patients at risk table is attached below the curves. (G) As in (F), but in RG dataset 2.

The enrichment analysis of the differentially expressed genes revealed that the three immune subtypes were active for distinct molecular functions (Fig. 3A). Pathways involved in antigen presenting, extracellular matrix remodeling, cell proliferation, and cell migration were upregulated in the inflamed subtype C3. In contrast, the aforementioned pathways were downregulated in the ”immune‐cold” subtype C2. The subtype was active for calcium and potassium signaling, endocytosis, and phagocytosis, as well as the maintenance of normal brain functions. Subtype C1, on the other hand, was in an intermediate state between C2 and C3, it might be in the transition from the immune‐dessert phenotype to the inflamed phenotype.

The normalized enrichment scores for the hallmark gene sets were calculated to validate the results of the DEG enrichment analysis (Fig. 3B). Together, the results showed that the inflamed subtype upregulated molecular functions that underpin various immune escape mechanisms involving innate immune suppression, hypoxia, and the remodeling of ECM (extracellular matrix, Fig. 3A,B). To examine whether the three immune subtypes tended to employ different immune escape mechanisms, we calculated the single‐sample enrichment scores of pathways that associate with immune escape (Fig. 3C). Consistent with its ”immune‐cold” phenotype, subtype C2 downregulated leukocyte adhesion, and suppressed antigen presentation. In contrast, the inflamed subtype C3 was enriched for tumor‐infiltrating lymphocytes, including immunosuppressive MDSCs, M2 macrophages, and regulatory T cells. Notably, upregulated angiogenesis gave rise to aberrant vasculature and high tumor oxygen consumption, which led to tissue hypoxia. Together with immunosuppressive cytokines secreted by tumor‐associated macrophages, hypoxia activated the STAT3 pathway, triggering hypoxia‐induced immunosuppression in subtype C3. Subtype C1 maintained high activity of calcium signaling as subtype C2, but the initiation of ECM remodeling, lymphocyte infiltration, antigen presentation, and hypoxia was also evident.

The difference in immune cell infiltration between glioma immune subtypes was not only confirmed by transcriptomic‐based estimation (Fig. S2), but also verified by H&E‐stained histological images in RG dataset 1 and RG dataset 2 (Fig. 3D). Immune escape often led to poor patient prognosis. Consistently, Kaplan–Meier survival analysis showed that the immune subtypes had significantly different patient survival (log‐rank *P* = 0.0001, Fig. 3E). Patients in the ”immune‐cold” subtype showed the longest mean OS (45.6 ± 4.9 months, *n* = 55), far exceeding the mean OS of the inflamed subtype (25.7 ± 3.2 months, *n* = 61, *P* = 0.0001). The mean OS of patients in subtype C1 reached 35.3 ± 3.5 months (*n* = 94). The survival benefit of the “immune‐cold” subtype and the poor OS of the inflamed subtype were also confirmed in RG dataset 1 and 2 (Fig. 3F,G). Moreover, the PFS of the patients following radiation therapy (Table 1) were collected for patients in RG datasets 1 and 2. Consistently, survival benefit was observed for the “immune‐cold” subtype, the patients had the longest PFS (18.1 ± 2.6 months in RG dataset 1, 23.7 ± 1.8 months in RG dataset 2), which was significantly better than the mean PFS of subtype C1 (12.1 ± 2.6 months in RG dataset 1, 15.1 ± 3.0 months in RG dataset 2) and C2 (3.7 ± 0.1 months in RG dataset 1, 7.9 ± 2.4 months in RG dataset 2).

The immune subtypes have not only shown distinct patient prognoses but also exhibited varied potential in immunotherapy responsiveness. Tregs were known to suppress effective tumor immunity, whereas macrophages were shown to suppress T‐cell recruitment. Both Tregs and macrophage subtypes were targeted in studies for effective immunotherapy. As shown in Fig. 2G, we observed that subtype C3 showed a significantly higher level of Treg infiltration (0.7 ± 0.1, *P* < 0.0001), whereas Treg infiltration in subtype C2 was minimal (0.2 ± 0.1; 0.4 ± 0.1 for C1). Similarly, subtype C3 exhibited significantly increased macrophage (0.6 ± 0.1, *P* < 0.001) and monocyte infiltration (0.6 ± 0.1, *P* < 0.001) compared to subtype C2 (macrophage 0.2 ± 0.1, monocyte 0.5 ± 0.1) and C1 (macrophage 0.4 ± 0.1, monocyte 0.5 ± 0.1). These differences were validated by two immune deconvolution methods, and were also replicated in multicenter datasets. The ICP blockade proteins are the most promising targets for cancer immunotherapeutic treatments. Our data showed that subtype C3 showed significantly higher expression of PD‐1 signaling participants (including PDCD1 and CD274, Fig. S3A), CTLA4 inhibitory signaling participants (including CTLA4, CD80, and CD86, Fig. S3B), and major ICP genes (including LAG‐3, Fig. S4). The results suggest that subtype C3 is more likely to respond to immune checkpoint inhibitors.

### 3.4 Radiomic signature serves as a noninvasive approach to predicting glioma immune subtypes

**Fig. 4:**
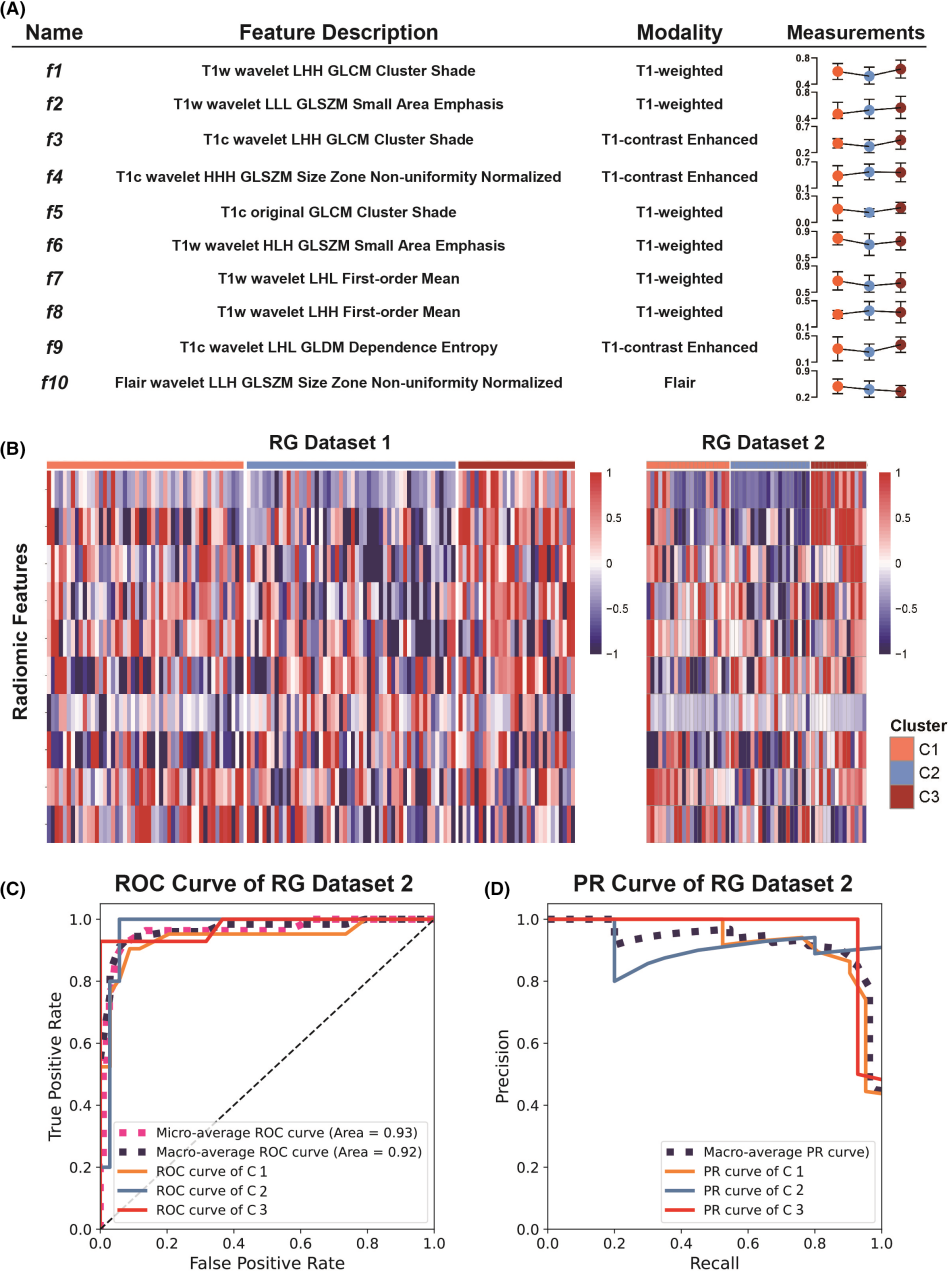
The validated immune subtypes can be predicted by a radiomic signature. (A) The name, feature description, and quantitative measurements of the 10 radiomic features (f1‐f10). The measurements for subtype C1 (orange), C2 (blue), and C3 (brick) are color coded. (B) Heatmap showing the quantitative values of the 10 radiomic features varied in the three immune subtypes. The same trend was observed in RG dataset 1 and RG dataset 2. (C) Receiver operator characteristic (ROC) curve showing the predictive power of the radiomic signature in RG dataset 2 (*n* = 21 for C1, *n* = 20 for C2, and *n* = 14 for C3). (D) Precision‐recall (PR) curve showing the predictive power of the radiomic signature in RG dataset 2.

Next, we sought to develop a noninvasive and financially affordable approach to predicting glioma immune subtypes. In RG dataset 1, a total of 3404 radiomic features were retrieved from the tumor region in MRI. Robust feature selection yielded 10 radiomic features whose values were statistically different among three subtypes (Fig. 4A,B). A detailed description of the visual properties of the 10 radiomic features can be found in Table S3. A radiomic signature constituted with the 10 radiomic features was built on RG dataset 1 to predict the immune subtype. The performance of the radiomic signature was assessed in RG dataset 2, showing an accuracy of 0.909, AUC of 0.924 (macro average, 95% CI: 0.889–1), and an F1 score of 0.905 (The ROC curve and precision‐recall curve is plotted in Fig. 4C,D, respectively).

### 3.5 Predictive radiomic features correlated with key biological processes that were associated with tumor immune escape

**Fig. 5:**
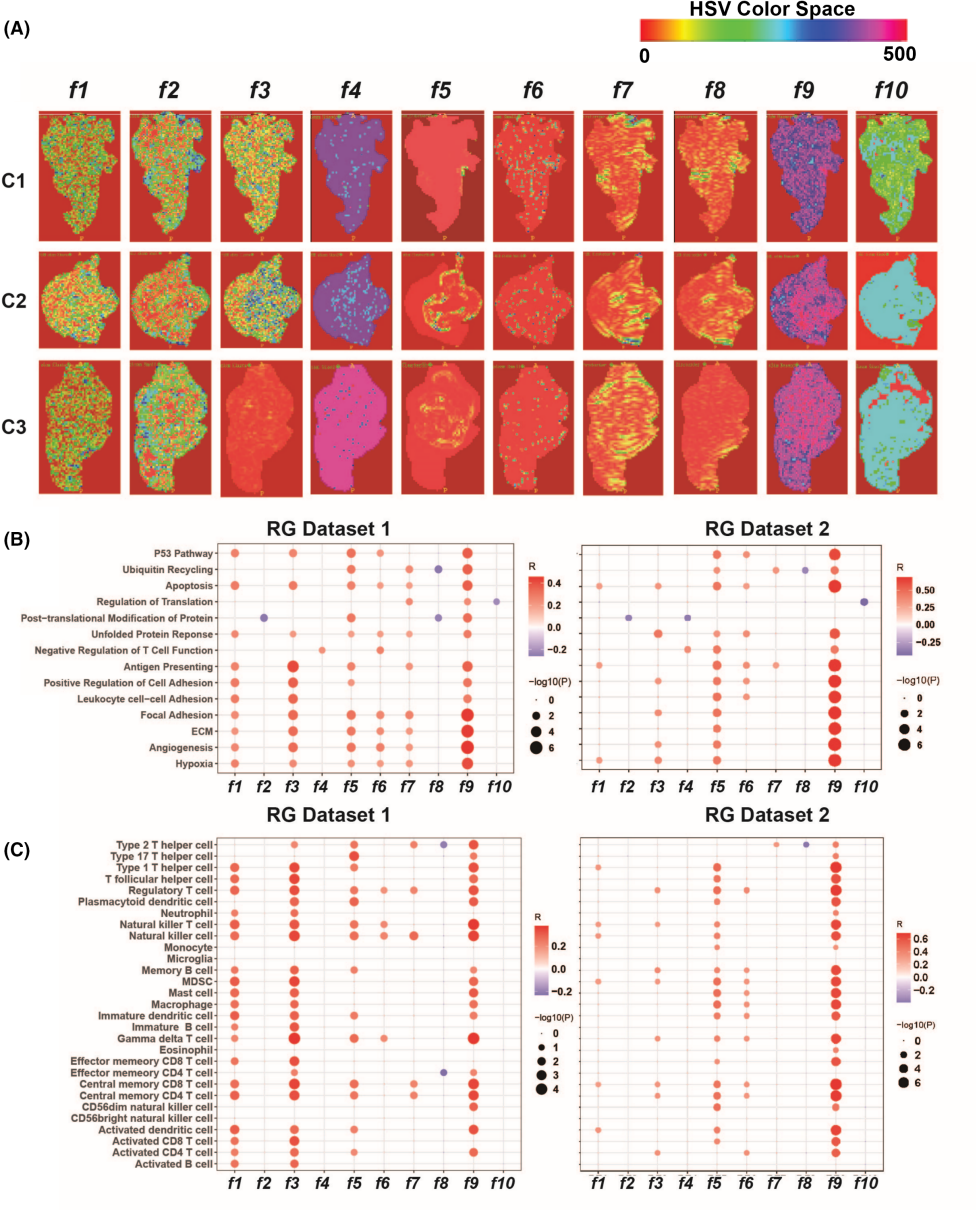
Ten radiomic features were significantly correlated with key biological functions and immune cell infiltration. (A) Feature maps delineating visual properties of the 10 radiomic features (f1‐f10). One sample was selected from each subtype (C1, C2, and C3). The tumor regions on MR images of the samples are visualized by the software “ITK‐SNAP.” Radiomic features were extracted from the tumor regions by the “PyRadiomic” package. Pseudo‐color was applied to the radiomic features; the features are shown with the HSV (hue, saturation, value) colormap. (B) Correlation between radiomic features and biological functions is shown in the bubble plots. The correlations were calculated by Pearson's correlation analysis. The sizes of the bubbles denote the FDR adjusted *P* values, whereas the color of the bubbles corresponds with the correlation coefficient *R*. The left panel shows the results in RG dataset 1, whereas the right panel shows the results in RG dataset 2. (C) As in (B), but showing the correlation between radiomic features and the abundance of immune cell infiltration.

The feature maps of the 10 radiomic features were calculated and are visualized in Fig. 5A. We observed that features 3, 4, 8, 9, and 10 manifested vast differences across the entire tumor region among subtypes, whereas features 1, 2, 5, 6, and 7 highlighted subtle variations in texture and intensity on the periphery of the tumor or within the tumor core. These radiomic features seemed to depict morphological and structural characterizations of the tumors and their vasculature. Indeed, tumor immune escape was underpinned by aberrant cell proliferation, metastasis, and angiogenesis. These biological processes could shape the tumor, and may result in diverse imaging phenotypes.

To investigate whether these radiomic features were associated with biological functions specific to tumor growth and immunity, we calculated the single‐sample enrichment scores of biological pathways supporting tumor immune escape, tumor hallmark, and general biological processes in RG dataset 1. Pearson's correlation analysis demonstrated that 10 radiomic features significantly correlated with biological pathways that were involved in programmed cell death (P53 pathway, ubiquitin recycling, and apoptosis), protein processing (regulation of translation, post‐translational modification, and unfolded protein response), immunity (regulation of T‐cell, antigen‐presenting), cell adhesion (regulation of cell adhesion, leukocyte adhesion, and focal adhesion), ECM remodeling, hypoxia, and angiogenesis (Fig. 5B, left panel).

To validate whether the correlation between the 10 radiomic features and these biological functions could be replicated, we extracted the 10 radiomic features from the MRI sequences of the patients in RG dataset 2. Enrichment scores were calculated for the identified biological pathways, and Pearson's correlation analysis confirmed that these pathways were significantly correlated with one to five radiomic features each (Fig. 5B, right panel).

Considering that the immune subtypes were identified based on immune cell infiltration, we calculated the Pearson's correlation coefficient between estimated immune cell abundance and the radiomic features. It was shown that the expression of 25 types of immune cells significantly correlated with two to six radiomic features in RG dataset 1 (Fig. 5C, left panel). The strong correlation underlies the biological interpretability of the radiomic signature for the prediction of glioma immune subtypes.

### 3.6 The reproducibility of the findings was verified in three independent datasets

To examine the reproducibility of the findings, we repeated our study in multicenter datasets. First, the TIL estimation and unsupervised clustering approach were applied to three multicenter datasets, and generated the same three immune subtypes (Fig. 2A). The reproducibility of clusters across the SD dataset and RG datasets were evaluated by IGP statistics. IGP showed high consistency for three clusters, with IGP values of 100% for C2 and C3 in both RG datasets, 87.8% for C1 in RG dataset 1, and 90.5% for C1 in RG dataset 2. The subtypes exhibited similar molecular features in three datasets (Fig. 3). Statistical differences in TIL between subtypes were also confirmed in the three datasets (Fig. 2G, Fig. S2). The gene expression pattern and significantly enriched pathways of the three immune subtypes were identified in the SD dataset and validated in RG datasets 1 and 2 (Fig. 3A–C, Fig. S2). Moreover, statistical differences in patient OS and PFS were also confirmed in three datasets (Fig. 3E–G). Second, to predict the immune subtypes by MR images, a radiomic signature was established in RG dataset 1, and was validated in RG dataset 2 (Fig. 4B,C). Statistical correlations between the radiomic features and key biological pathways were identified and verified in RG dataset 1 and RG dataset 2, respectively (Fig. 5B). Furthermore, the significant correlations between radiomic features and TILs were reproduced in RG dataset 2. The expression of 24 types of immune cells significantly correlated with the quantitative values of one to five radiomic features (Fig. 5C, right panel).

## 4 Discussion

The immune landscape of glioma has gained broad attention, as it was considered a substantial piece of the puzzle that may explain why immunotherapy failed to trigger clinical responses in patients with glioma. The main contribution of the study was to identify three glioma immune subtypes that employed various immune escape strategies, and to develop a noninvasive approach to predict the immune subtype of glioma with preoperative MR images. The key strengths of the study lie in three aspects: first, the identification of glioma‐specific immune subtypes using a customized immune signature panel in adult diffuse gliomas; second, the establishment of a radiomic signature that enables the noninvasive prediction of immune subtypes, and the verification of the biological interpretability of the radiomic signature; and third, the reproducibility of the major findings that was revealed by multicenter datasets.

The immune subtype of malignant gliomas had been extensively studied. Wang *et al*. [13] identified four distinct GBM immune subtypes; one subtype showed overall higher immune infiltration scores, two subtypes exhibited reciprocal ratios of macrophages and lymphocytes, whereas the fourth subtype contained substantially lower enrichment for all immune cell types. Similarly, three glioma immune subtypes were discovered and validated in the TCGA and CGGA cohorts [22]. Subtype 1 represents “hot” tumors, whereas subtype 2 and 3 were immunologically “cold” tumors. Unlike the published studies that applied pan‐cancer immune gene signatures to estimate immune cell infiltration, we tailored the signature gene sets to include brain resident microglia, thus establishing a glioma‐specific scoring method for the estimation of immune cell infiltration. To the best of our knowledge, this is the first study that identified immune subtypes for adult diffuse gliomas with a brain TME‐specific immune gene signature panel. Moreover, to eliminate the impact of cohort constitution on unsupervised clustering, we included study cohorts that resembled the partitioning of grade 2–4 patients in the real world, where GBM accounts for 60% of all gliomas [2, 3]. Our data suggested that immune subtype C2 is favored in patients of grade 2 and 3, whereas subtype C3 is more common in patients with grade 4 tumor. However, none of the immune subtype is predominant in grades 2, 3, or 4 tumors. Therefore, we believe that the immune subtypes represented a unique classification across grades of adult‐type diffuse glioma.

We identified three glioma immune subtypes that exhibited enriched, medium, and scarce immune cell infiltration. The inflamed subtype C3 was enriched in both antitumor immune cells and protumor immune cells. Intriguingly, angiogenesis, hypoxia, and interleukin‐STATs signaling pathways were upregulated in subtype C3. These observations substantiate the hypothesis that tumors of subtype C3 were marked by angiogenesis‐induced hypoxia and elevated cytokine secretion by TAMs, which in turn activates STATs signaling and triggers immune suppression [40]. In addition, the remodeling of ECM and enhanced leukocyte adhesion favored the infiltration of immune cells including regulatory T cells and MDSCs, which further enhanced the immunosuppressive microenvironment. In contrast, the “cold” subtype C2 employed a very distinctive immune escape mechanism. ECM remodeling and leukocyte adhesion were downregulated, which blocked the infiltration of all immune cells. Meanwhile, antigen presentation was downregulated, which reduced the chance of initiating immune responses. On the other hand, subtype C2 attempts to maintain brain cell functions, as well as substrate transportation and calcium signaling. Subtype C1 appeared to represent a transient state between the inflamed and “cold” phenotype, where calcium signaling was still upregulated, but angiogenesis‐induced hypoxia and immune cell infiltration were also elevated. It was evident that the three immune subtypes employed different immune escape strategies. For inflamed subtype C3, combined treatment of immunotherapy and targeted therapy may alleviate angiogenesis and immune check‐point blockage. In contrast, these regimens may not trigger clinical responses in subtype C2.

To develop a noninvasive imaging tool for the identification of glioma immune subtypes, we constructed a radiomic signature that could achieve a prediction accuracy of 0.909. This noninvasive approach allows the identification of glioma immune subtypes at diagnosis. More important, it could be applied to monitor the TME throughout the course of the disease. The immune landscape of tumor undergoes dynamic changes [41], which in turn highlights the importance of immune surveillance. Facing the ethical dilemma of experimenting with human brain tissues, it is not possible to obtain brain tissues repetitively to monitor the molecular alterations in gliomas. To address this practical challenge, multiple studies have established radiomic signatures to predict the molecular feature of tumors [42, 43] and their responses to various treatments [23, 44]. Consistently, we established a radiomic signature that enables dynamic monitoring of the glioma immune landscape throughout the disease course. The immune subtype of glioma provides information on the molecular characterization and potential drug target of the tumor, which allows prompt adjustment of treatment strategies at all times.

The lack of biological interpretability posed barriers to the clinical application of machine‐learning‐based prediction models. To explore the biological meaning of radiomic features forming the radiomic signature, we assessed the correlation between the radiomic features and various cellular and molecular functions. Intriguingly, the radiomic features were significantly associated with pathways involved in immune responses, ECM remodeling, angiogenesis, and hypoxia. Most of the radiomic features directly correlated with immune cell infiltration. The radiomic features also correlated with essential cellular functions including programmed cell death and protein processing. Therefore, it could be postulated that the immune subtypes that employed distinct immune escape strategies may exhibit different tumor morphologies, vasculature, encapsulation, and stromal morphology. These alterations can be captured by MR images, and were converted to changes in intensity and texture shown by radiomic features.

To verify the reproducibility of the findings, we replicated the studies in three independent datasets. The immune subtypes of glioma were validated at four levels. First, the TILs were calculated and cross‐validated using three algorithms. Second, unsupervised clustering was performed in three datasets, and the same three immune subtypes were yielded. Third, the statistical difference in TIL and the molecular characterizations of the three immune subtypes were confirmed in three datasets. Fourth, the statistical differences in patient prognoses were also confirmed in three datasets. In terms of immune subtype prediction, the predictive power of the radiomic signature was examined in RG dataset 1 and RG dataset 2. Moreover, the radiomic features forming the radiomic signature were significantly associated with key immune escape pathways and the abundance of TILs. These correlations were discovered in RG dataset 1, and were validated in RG dataset 2. The repetitive validation of the findings in multicenter datasets revealed the strong reproducibility of the results, which has incrementally strengthened the credibility of the major findings.

Our study has three major limitations. First, the retrospective nature and the relatively small size of the datasets impaired the strength of the conclusions, although multicenter datasets were used to enhance the statistical power. Second, the impact of the glioma immune subtype on immunotherapy was hypothetical. Clinical trials are required to examine whether the immune subtype could guide precision immunotherapy. Third, future experiments and prospective studies are required to dissect the mechanisms in depth.

## 5 Conclusion

In conclusion, three immune subtypes were identified and validated in multicenter datasets diagnosed with adult‐type diffuse glioma. The inflamed, intermediate, and “cold” subtypes exhibited distinct immune escape mechanisms, based on which personalized treatment regimens can be designed. A radiomic signature was constructed to predict glioma immune subtypes noninvasively. The biologically interpretable signature achieved a high prediction accuracy, which enables the surveillance of immunological dynamics throughout the course of the disease. The major findings were validated in multicenter datasets, which guaranteed the reproducibility of the results. In the future, a larger clinical dataset should be collected to strengthen the power of the predictive radiomic signature. Clinical trials should be initiated to examine whether personalized immunotherapy guided by the immune subtypes would effectively prolong patient survival.

## Conflict of interest

The authors declare no conflicts of interest.

## Author contributions

JD, ZZ, and YC contributed equally to this work; JD, ZL, ZZ, JC, and JY designed the study. ZZ, YC, JC, WW, and JY collected clinical samples, and were responsible for patient management. JD, ZZ, QS, and YZ performed data analysis. HZ and DL were responsible for project management and funding acquisition. All authors were involved in the construction of the article. All authors approved the final version of submitted article. JD, JY, ZZ, and ZL proof read the article.

### Peer review

The peer review history for this article is available at https://publons.com/publon/10.1002/1878‐0261.13380.

## Supporting information


**Fig. S1.** RNA sequencing depth and K number determination.
**Fig. S2.** Immune subtypes were validated in RG dataset 1 and 2.
**Fig. S3.** Gene expression level of ICP signaling participants in three immune subtypes.
**Fig. S4.** ICP gene expression in three immune subtypes.

**Table S1.** Patient Inclusion and Exclusion Criteria.

**Table S2.** Gene sets of transcriptomic signatures.

**Table S3.** Description of radiomic features.

## Data Availability

The data that support the findings of this study are openly available in Chinese Glioma Genome Atlas (CGGA) at http://www.cgga.org.cn/ under the dataset ID: mRNAseq_693; and Science Data Bank (ScienceDB) at http://www.cgga.org.cn/ under the data DOI: 10.57760/sciencedb.07164. The rest of the supporting materials are available from the corresponding authors upon request.
