# Supplementary material for: Imaging phenotypes from MRI for the prediction of glioma immune subtypes from RNA sequencing: A multicenter study
Source: Mol Oncol. 2023 Feb 12;17(4):629–46. doi: 10.1002/1878-0261.13380 (PMC10061289; doi:10.1002/1878-0261.13380)
Supplement: Supplementary file 1 — Fig. S1. RNA sequencing depth and K number determination. Fig. S2. Immune subtypes were validated in RG dataset 1 and 2. Fig. S3. Gene expression level of ICP signaling participants in three immune subtypes. Fig. S4. ICP gene expression in three immune subtypes. [file MOL2-17-629-s004.pdf]

# Supplementary Figures

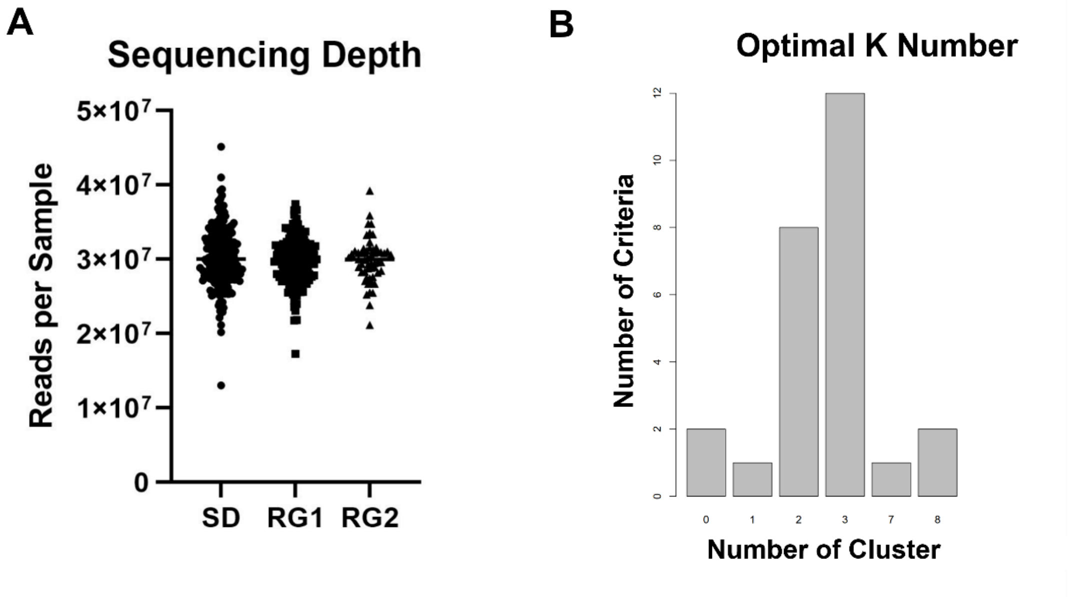


**Supplementary Figure 1 RNA sequencing depth and K number determination**
**(A)** RNA sequencing depth of each individual in the three datasets was shown in the scattered plot. Data were presented as reads per sample. **(B)** The optimal K number for K-means clustering was determined using NBcluster R package.

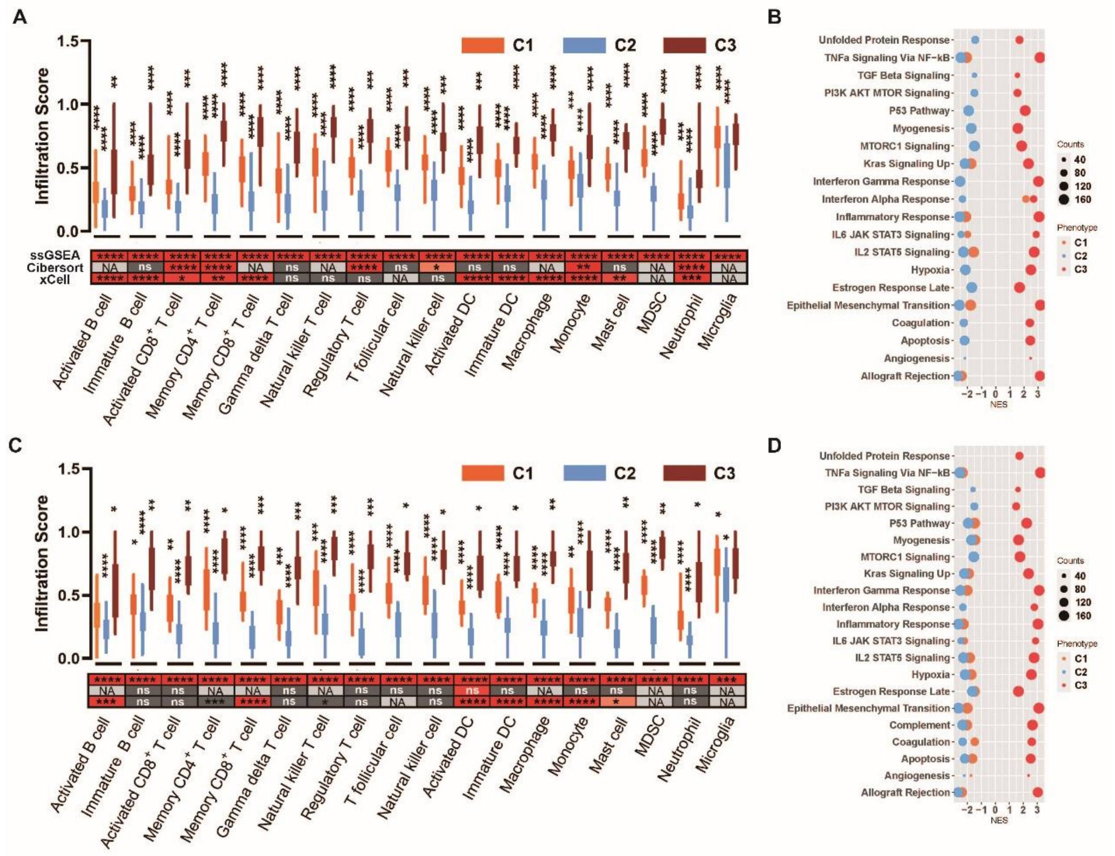


**Supplementary Figure 2 Immune subtypes were validated in RG dataset 1 and 2**

**(A)** Boxplots showing the infiltration scores of 18 types of immune cells that were differentially enriched among the three immune subtypes in RG dataset 1, n=130. Statistical comparisons were conducted with one-way ANOVA or Krustal-Wallis test depending on the normality of the datasets. P values were calculated with the chi-square test. * for p<0.05, ** for p<0.01, *** for p<0.001, **** for p<0.0001. * above orange boxes shows comparison between C1 and C2, * above blue boxes shows comparison between C2 and C3, * above brick boxes shows comparison between C1 and C3. The immune cell infiltration was calculated by ssGSEA, Cibersort, and xCell separately, the p values resulting from statistical comparisons between the three subtypes were displayed in the heatmap. **(B)** The normalized enrichment scores of the three subtypes for GSEA hallmark gene sets were shown in the bubble plot, the results were for RG dataset 1. The size of the bubbles denotes the counts of genes significantly enriched for each pathway. The subtypes were color-coded (orange for C1, blue for C2, and brick for C3). **(C)** As in (A), but for RG dataset 2, n=55. **(D)** As in (B), but for RG dataset 2.

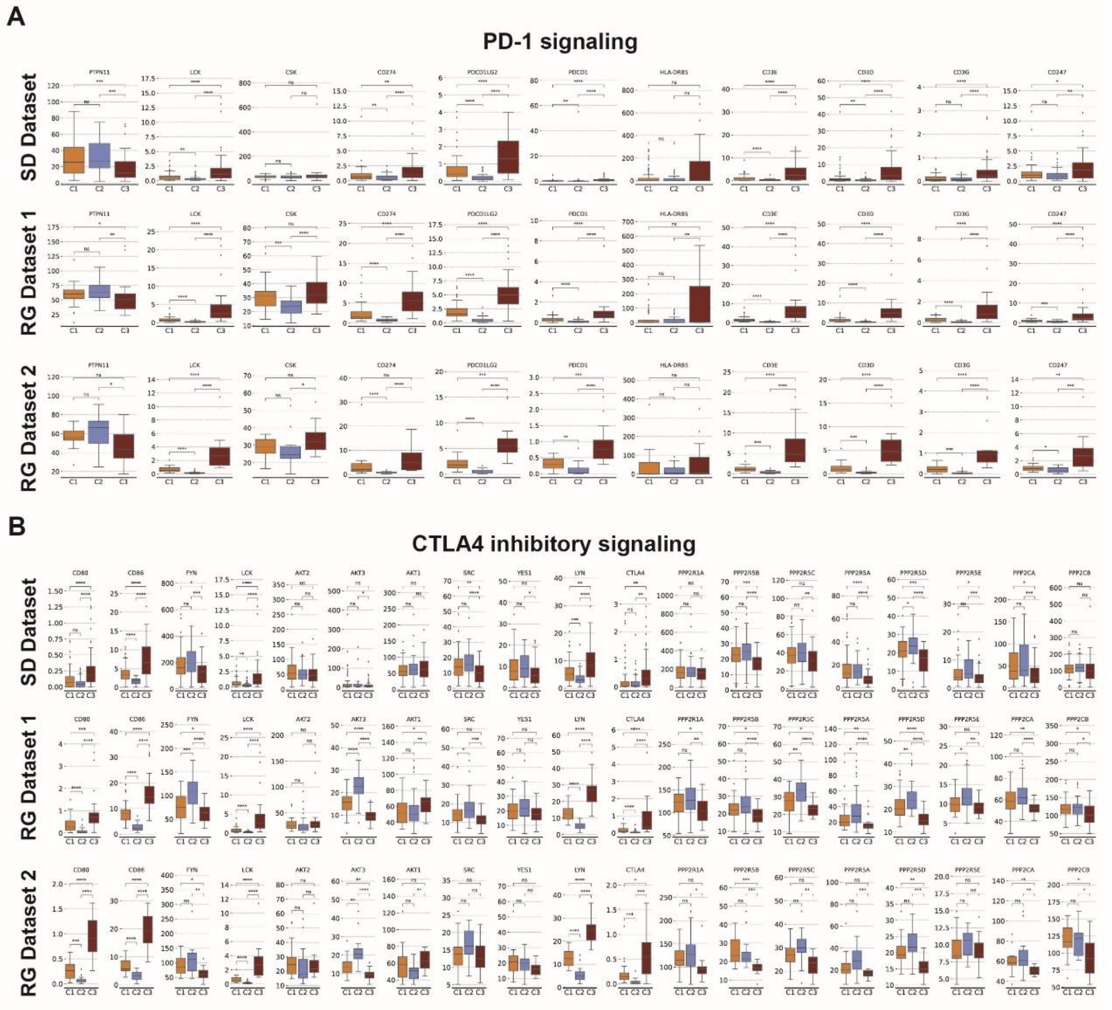


**Supplementary Figure 3 Gene expression level of ICP signaling participants in three immune subtypes**

PD-1 signaling pathway (A) and CTLA-4 inhibitory pathway (B) participants were obtained from the Reactome database. The RNA expression level of the genes were compared between three immune subtypes. Statistical differences between groups were compared by one-way ANOVA or Krustal-Wallis test depending on the result of normality tests and variation tests, and were followed by Bonferroni correction. P values were denoted by stars, * for $p<0.05$, ** for $p<0.01$, *** for $p<0.001$, **** for $p<0.0001$, ns for not significantly different. n=210 for SD dataset, n=130 for RG dataset 1, and n=55 for RG dataset 2.

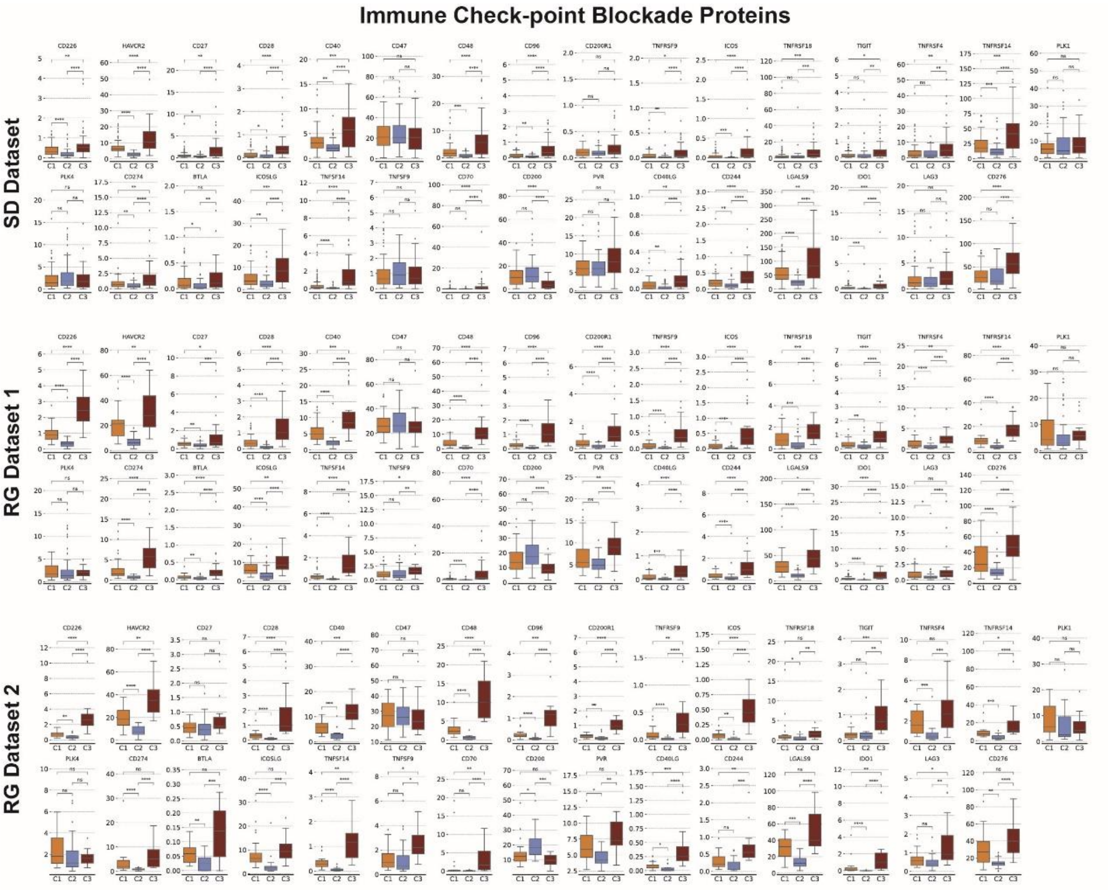


**Supplementary Figure 4 ICP gene expression in three immune subtypes**

The RNA expression level of immune checkpoint (ICP) blockade proteins were compared between three immune subtypes. Statistical differences between groups were compared by one-way ANOVA or Krustal-Wallis test depending on the result of normality tests and variation tests, and were followed by Bonferroni correction. P values were denoted by stars, * for p<0.05, ** for p<0.01, *** for p<0.001, **** for p<0.0001, ns for not significantly different. n=210 for SD dataset, n=130 for RG dataset 1, and n=55 for RG dataset 2.
